# A Novel Plasmid DNA-Based Foot and Mouth Disease Virus Minigenome for Intracytoplasmic mRNA Production

**DOI:** 10.3390/v13061047

**Published:** 2021-06-01

**Authors:** Ploypailin Semkum, Challika Kaewborisuth, Nattarat Thangthamniyom, Sirin Theerawatanasirikul, Chalermpol Lekcharoensuk, Payuda Hansoongnern, Pongrama Ramasoota, Porntippa Lekcharoensuk

**Affiliations:** 1 Interdisciplinary Graduate Program in Genetic Engineering, The Graduate School, Kasetsart University, Bangkok 10900, Thailand; ploypailinvet69@gmail.com; 2 Department of Microbiology and Immunology, Faculty of Veterinary Medicine, Kasetsart University, Bangkok 10900, Thailand; Golf_Zealand@hotmail.com (N.T.); kungwan29@hotmail.com (P.H.); 3 Center for Advanced Studies in Agriculture and Food, KU Institute for Advanced Studies, Kasetsart University, Bangkok 10900, Thailand; 4 Virology and Cell Technology Research Team, National Center for Genetic Engineering and Biotechnology, National Science and Technology Development Agency, Pathum Thani 12120, Thailand; Challika.kae@biotec.or.th; 5 Department of Anatomy, Faculty of Veterinary Medicine, Kasetsart University, Bangkok 10900, Thailand; fvetsrth@ku.ac.th; 6 Department of Companion Animal Clinical Sciences, Faculty of Veterinary Medicine, Kasetsart University, Bangkok 10900, Thailand; fvetcpl@ku.ac.th; 7 Department of Social and Environmental Medicine, Faculty of Tropical Medicine, Mahidol University, Bangkok 10400, Thailand; pongrama.ram@mahidol.ac.th

**Keywords:** picornaviruses, *foot and mouth disease virus* (FMDV), reverse genetics, minigenome, replicon, vaccine, antiviral drug screening

## Abstract

Picornaviruses are non-enveloped, single-stranded RNA viruses that cause highly contagious diseases, such as polio and hand, foot-and-mouth disease (HFMD) in human, and foot-and-mouth disease (FMD) in animals. Reverse genetics and minigenome of picornaviruses mainly depend on in vitro transcription and RNA transfection; however, this approach is inefficient due to the rapid degradation of RNA template. Although DNA-based reverse genetics systems driven by mammalian RNA polymerase I and/or II promoters display the advantage of rescuing the engineered FMDV, the enzymatic functions are restricted in the nuclear compartment. To overcome these limitations, we successfully established a novel DNA-based vector, namely pKLS3, an FMDV minigenome containing the minimum cis-acting elements of FMDV essential for intracytoplasmic transcription and translation of a foreign gene. A combination of pKLS3 minigenome and the helper plasmids yielded the efficient production of uncapped-green florescent protein (GFP) mRNA visualized in the transfected cells. We have demonstrated the application of the pKLS3 for cell-based antiviral drug screening. Not only is the DNA-based FMDV minigenome system useful for the FMDV research and development but it could be implemented for generating other picornavirus minigenomes. Additionally, the prospective applications of this viral minigenome system as a vector for DNA and mRNA vaccines are also discussed.

## 1. Introduction

The members of *Picornaviridae* including, *foot-and-mouth disease virus* (FMDV), *human enterovirus* 71, and *hand, foot-and-mouth disease virus*, are positive-sense single-stranded RNA viruses. The genomic RNA of approximately 8500 nucleotides is enclosed within a non-enveloped capsid with an icosahedral structure [1,2]. In infected cells, the viral RNA genome encodes only a single polyprotein, which is post-translationally processed by viral proteases to yield three intermediate protein precursors (P1-3). The intermediate precursors are sequentially cleaved by viral proteases (L^pro^ and 3C^pro^) into thirteen mature viral proteins [3,4]. The P1 protein precursor encodes four structural proteins, VP1-4, to form the viral capsid, while L protein and those encoded by the P2 (2A, 2B, 2C) and P3 (3A, 3B, 3C, and 3D) regions are non-structural proteins [5]. The coding region is flanked by untranslated regions (UTRs) at 5′ and 3′ends, so-called 5′UTR and 3′UTR, respectively, and downstream of the 3′UTR is a poly A tail. These non-coding regions are the crucial cis-acting elements involved in viral replication, transcription, and translation.

The 5′UTR of picornaviruses ranges from 415 to 1451 nucleotides, depending on viral species. It forms a complex secondary structure involved in viral replication and translation [6]. FMDV 5′UTR is approximately 1 kb, which consists of an S-fragment, a poly C tract, pseudoknots, a cis-acting replication element (cre), and an internal ribosome entry site (IRES) [3,6]. The S-fragment is approximately 360 nucleotides folding into a long stem–loop which function is undetermined. Following the S-fragment is a poly C tract containing a long stretch of variable cytosine (C) residues [7,8]. A minimum of 6 C residues is required to maintain viral infectivity [8]. The downstream region of the poly C tract contains the putative series of RNA pseudoknots with unknown functions. The cre containing a conserved AAACA motif serves as a template for 3D^pol^-mediated uridylylation of 3B [2], later renamed as 3B-uridylylation site (bus). All picornavirus 5′UTRs contain an IRES for cap-independent translation initiation. It recruits the ribosomes for assembly and scanning to the start codon which is facilitated by cellular factors known as IRES trans-acting factors (ITAFs) [9]. Thus far, five groups of picornavirus IRESs have been identified based on the physical and biological properties. The FMDV IRES is classified as a type II IRES [10].

The 3′UTR also plays a role in the functional IRES element. It acts as a translation enhancer to mediate the initiation of cap-independent translation of several plants’ RNA viruses [11]. Moreover, FMDV 3′UTR specifically interacts with the 5′UTR cis-acting element to regulate viral replication [12]. Picornavirus 3′UTR contains two stable stem–loops that are involved in the negative-strand synthesis of viral RNA [12,13] by serving as a template for uridylylated 3B to initiate viral RNA replication by 3D^pol^ [14]. The downstream 3′UTR contains a poly A tail with variable lengths, which is crucial for the negative-strand synthesis. The poly A tail with a length lesser than eight residues has been shown to suppress polioviral replication [15] due to insufficient length for the 3D^pol^ binding [16]. The 3′UTR with poly A tail also stabilizes viral RNA and controls the translation process. Moreover, the protein translation activity of each viral 5′UTR appeared to be enhanced by the cognate 3′UTR [17]. Additionally, the length of poly A tail drastically influenced translation efficiency in duck hepatitis A virus type 1 [14].

Reverse genetics of picornaviruses, including FMDV, mainly depends on in vitro transcription and RNA transfection [1,18,19,20]. RNAs were synthesized in vitro by T7 or SP6 RNA polymerases using cDNA containing the whole genome as a template [18,21,22]. In addition, in vitro transcribed FMDV replicons with reporter genes have been developed for molecular virological studies [23,24,25,26]. However, the major disadvantages of this reverse genetics system and the replicons are instability of the synthesized RNA and lower infectivity of the recombinant viruses compared with their parental viruses. Recently, RNA polymerase I (Pol I)-based reverse genetics system [27] and Pol I-Pol II system [13,28] have been described for rescuing the engineered FMDV. Both mammalian Pol I and Pol II promoters perform their functions in the intranuclear compartment.

Herein, we have proposed the establishment of a plasmid DNA-based FMDV minigenome that can efficiently generate uncapped mRNAs within the cytoplasmic compartment. This imitates the generation of a viral genomic RNA-like molecule, which can be used as a template for the cellular translation process. In this study, our FMDV minigenome together with the helper plasmids could proficiently drive the transcription and translation of the green fluorescent protein (GFP) gene in Baby Hamster Kidney-21 (BHK-21) cells. We have also demonstrated the application of the FMDV minigenome for cell-based antiviral drug screening. In a parallel study, this minigenome carrying the full FMDV polyprotein coding region, when transfected with the two helper plasmids, could produce infectious FMDVs. We believe that not only is this system suitable for FMDV but it can also be applied for other picornavirus minigenomes. Moreover, it can be used for foreign protein expression such as a DNA vector but working solely in the cytoplasm.

## 2. Materials and Methods

### 2.1. Cells and Viruses

BHK-21 cells (ATCC) were cultured in Minimum Essential Medium (MEM, Invitrogen™, Waltham, MA, USA) supplemented with 10% fetal bovine serum (FBS) (Invitrogen™, Waltham, MA, USA), 100 IU/mL ampicillin, and 2 mM L-glutamine and incubated at 37 °C with 5% CO_2_. The FMDV used in this study was provided by the Bureau of Veterinary Biologics, DLD, Thailand, including serotype A; A/Lopburi/2012 (A/Lop), type O; O/TAI/189/1987 (O189), and type Asia1; Asia1/2013. All viruses were cultivated in BHK-21 cells, which were maintained in MEM supplemented with 2% FBS.

### 2.2. RNA Isolation and cDNA Synthesis

Viral RNA was extracted using a viral nucleic acid extraction kit (Geneaid^®^, Taipei, Taiwan) following the manufacturer’s protocol. cDNA was generated from the RNA template using Superscript III RT (Invitrogen™, Waltham, MA, USA) according to the manufacturer’s instruction. Briefly, the reverse transcription (RT) reaction contained 200 ng of random hexamer primers, 1 µL of 10 mM dNTP, 1 µg of RNA, and dH_2_O up to 13 µL. The RT master mix consisting of 4 µL of 5× FS buffer, 1 µL of 0.1 mM DTT, 40 Units of RNase OUT, and 200 units of Superscript III RT was prepared before adding into the reaction tube. The reaction was incubated at 55 °C for 1 h prior to being stopped by incubation at 70 °C for 15 min.

### 2.3. FMDV Growth Kinetics Analysis

Growth kinetics of the FMDVs, including type A; A/Lopburi/2012, type O; O/TAI/189/1987, and type Asia1; Asia1/2013, were studied. Briefly, overnight seeded BHK-21 cells in 6-well plates were inoculated with each FMDV strain at a multiplicity of infection (MOI) of 0.01. Three replicates were included for each virus. After an hour of virus adsorption, the inoculum was then removed, and the cells were washed once with MEM and maintained in 2 mL of MEM containing 2% FBS. The infected cells were incubated at 37 °C with 5%CO_2_ for 24 h. The supernatants were collected at 6 different time points with 4-h intervals. The virus titers were calculated using the Reed and Muench method and reported as TCID_50_/_mL_.

The log10 of virus titers were analyzed for the difference between mean titers of all 6-time points of each isolate with the repeated measure ANOVA using the generalized linear model (GLM) method available in the SAS^®^ University Edition (SAS Institute Inc., 2015, Cary, NC, USA). Once the analysis showed at least one mean with a statistically significant difference, the post-hoc comparison with the Tukey method was applied to demonstrate the different one. In addition, the difference of mean titers at each time point was also evaluated with the GLM and Tukey post-hoc comparison. The *p*-value less than 0.05 was considered as a statistical significance. The viral growth kinetics were plotted between the virus titers and hours post-inoculation (hpi). The procedures containing live viruses were performed at the Bureau of Veterinary Biologics, DLD, Thailand.

### 2.4. Construction of FMDV Minigenome Vectors: pKLS3 and pKLS3_GFP

To generate the FMDV minigenome, the entire FMDV O189 UTR was PCR amplified, cloned, and sequenced. The 5′UTR fragment was divided into three overlapping DNA fragments—S-fragment, poly C tract, and the large fragment (l-fragment)—for amplification by fusion PCR. In the first reaction, the S-fragment, poly C, and l-fragment were amplified with each specific primer pairs (Table 1) using the corresponding plasmids as the templates. In the second reaction, the purified S-fragment and poly C tract were used as the templates for amplification to generate the fused S-poly C fragment. In the third reaction, the fused S-poly C DNA and l-fragment were used as the templates for amplification to produce the -full-length 5′UTR flanked by *Xho*I and *Stu*I recognition sequences.

**Figure 1:**
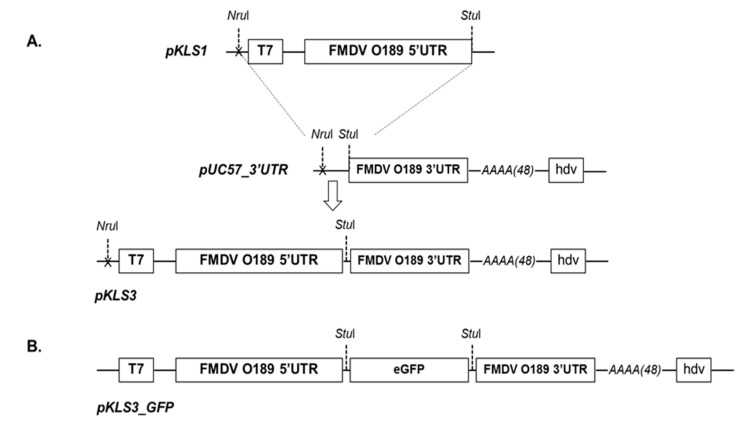
Construction of recombinant plasmids. (**A**) The DNA cassette containing T7 promoter and FMDV O189 5′UTR in pKLS1 vector was removed by digesting with the restriction enzymes, *Nru*I and *Stu*I, and placed upstream of FMDV O189 3′UTR, in plasmid pUC57_3′UTR, resulting in the pKLS3 vector. The complete pKLS3 construct is an FMDV minigenome containing the essential FMDV cis-acting elements for viral replication/transcription and translation and a *Stu*I site for foreign gene insertion. (**B**) The enhanced green fluorescence protein (GFP) gene was inserted into pKLS3 at the *Stu*I site between FMDV 5′and 3′UTRs to generate a minigenome with a transfection reporter pKLS3_GFP.

The pKLS3 vector (Patent Application Number: 1901006625) was modified from pKLS1. Briefly, the sequence of human polymerase I (Pol I) promoter was amplified using pDZ_NP2 [29] as the template and cloned into pGEM-T easy (Promega, Madison, WI, USA) downstream T7 RNA polymerase promoter resulting in pPol I. Subsequently, the full length 5′UTR of FMDV O189 was cloned into pPol I downstream of the Pol I promoter. In addition, the sequences of FMDV O189 3′UTR, a long poly A tail, and the hepatitis delta virus (hdv) ribozyme were synthesized (GenScript^®^, Piscataway, NJ, USA), which was subsequently cloned into the plasmid pUC57 vector (GenScript^®^, Piscataway, NJ, USA), resulting in pUC57_3′UTR. The DNA cassette containing the sequence T7 promoter-pol I promoter-FMDV O189 5′ UTR in 5′ to 3′ direction was excised with *Nru*I/*Stu*I restriction enzymes (New England Biolab, Ipswich, MA, USA) from the pKLS1 vector and then ligated into pUC57_3′UTR upstream the 3′UTR, to create pKLS3 vector (Figure 1A). The enhanced green fluorescence protein gene (*GFP*) was amplified from pEGFP-N1 (Takara Bio, Mountain View, CA, USA) by using primers EGFP_F and EGFP_R (Table 2). The GFP PCR product was purified and ligated into the pKLS3 vector at the *Stu*I restriction enzyme site, which is the junction between FMDV 5′ and 3′UTRs, resulting in pKLS3_GFP (Figure 1B).

### 2.5. Evaluation of Hepatitis Delta Virus Ribozyme (hdv) Activity by In Vitro Transcription

The activity of the hdv ribozyme was examined by in vitro transcription. The closed-circular plasmid DNA, pKLS, was used as the template for in vitro transcription with the application of the Riboprobe^®^-T7 in vitro Transcription Systems (Promega, Madison, WI, USA) to produce an RNA molecule containing the Pol I-FMDV_5′UTR-GFP-FMDV_3′UTR-poly A sequence with the length of approximately 2.2 kb. Briefly, 500 ng of the plasmid was mixed with 4 µL of the 5× Transcription Optimized Buffer, 0.1 M DTT, 20 U of Recombinant RNasin^®^ Ribonuclease Inhibitor, 2.5 mM of each rNTP, 15 U of T7 RNA Polymerase, and nuclease-free water to the final volume of 20 µL. The reaction was incubated at 37 °C for 1 h, and the RNA product was analyzed by agarose gel electrophoresis.

### 2.6. Construction of ‘Helper Plasmids’ for the FMDV Minigenome

The plasmid expressing T7 RNA polymerase, namely pCAGGS_T7, was constructed as described previously [30]. Briefly, the T7 RNA polymerase gene was amplified using DNA isolated from *E. coli* BL21 (DE3) (kindly provided by Prof. Dr. Wanpen Chaicumpa at Mahidol University, Thailand). The T7 RNA polymerase sequence was purified and cloned into pCAGGS to yield pCAGGS_T7. The sequence encoding the P3 open reading frame (ORF) of FMDV O189 was reverse transcribed using the viral RNA as the template by the action of Superscript III enzyme (Invitrogen, Waltham, MA, USA) with the random hexamer to generate the second helper plasmid. The cDNA was then served as the template to amplify the P3 DNA fragment using primers ClaI_P3_F and NheI_P3_R (Table 3). The FMDV P3 DNA fragment was purified using a gel purification kit (RBC Bioscience, Taipei, Taiwan) and cloned into the pGEM-T easy vector (Promega, Madison, WI, USA), resulting in pGEM_P3. The integrity of the nucleotide sequences in pCAGGS_T7 and pGEM_P3 were verified by DNA sequencing (Macrogen, Seoul, Korea). The fragment of FMDV P3 was double digested with *Cla*I/*Nhe*I restriction enzymes and ligated into the corresponding sites within the pCAGGS vector, resulting in pCAGGS_P3.

### 2.7. Transfection

The plasmids pKLS3_GFP, pCAGGS T7, and pCAGGS_P3, were purified using a Mini-Prep purification kit (GeneMark, Taichung, Taiwan), following the manufacturer’s protocol. In the co-transfection experiment, the pKLS3_GFP and pCAGGS_T7 were transfected directly into BHK-21 cells, while pKLS3_GFP, pCAGGS_P3, and pCAGGS_T7 were combined in the tri-transfection protocol. The combinations of plasmids were incubated with Lipofectamine™ 2000 transfection reagent according to the manufacturer’s instruction (ThermoFisher Scientific, Waltham, MA, USA) prior to dropping onto overnight seeded BHK-21 cells in 6-well plates. The protocol for co-transfection was optimized by varying the ratio of pKLS3_GFP to pCAGGS_T7 as 1:1, 1:2, and 1:3. In the co-transfection experiment, 10 µL Lipofectamine™ 2000 were incubated in a 250 µL Opti-MEM^®^ I reduced-serum medium (Invitrogen, Waltham, MA, USA) at room temperature for 5 min. Simultaneously, 1 µg of pKLS3_GFP and 1–3 µg of pCAGGS_T7 were equilibrated in 250 µL Opti-MEM^®^ I reduced-serum medium.

In the tri-transfection protocol, 1 µg of pKLS3_GFP, 3 µg of pCAGGS_T7, and 1 µg of pCAGGS_P3 were added to a microfuge tube containing 245 µL Opti-MEM^®^ I reduced-serum medium. The diluted DNA and transfection reagent were mixed with each other and incubated at room temperature for 20 min. Subsequently, the mixtures were gently added onto the monolayer of BHK-21 cells prior to incubation at 37 °C with 5% CO_2_ for 5 h. Then, the transfection medium was replaced with Opti-MEM^®^ I reduced-serum medium containing 2% FBS and the cells were incubated at 34 °C with 5% CO_2_. The mixture of pCAGGS_T7 and pCAGGS_P3 plasmids was also included as a negative transfection control. The co-transfection with pKLS3_GFP and pCAGGS_T7, and a single plasmid transfection with pKLS3_GFP were also performed in parallel to compare the expression level. After 24–48 h post-transfection (hpt), the cells were observed for green fluorescent signals using a fluorescence microscope (Olympus, Center Valley, PA, USA).

### 2.8. Inhibition of RNA Dependent RNA Polymerase (RdRp) Activities by Ribavirin

The tri-transfection of pKLS3 and the two helper plasmids was performed on a monolayer of BHK-21 cells in 6-well plates using the FuGENE^®^ HD transfection reagent (Promega, Madison, WI, USA) per the manufacturer’s instructions. Briefly, 1 µg of pKLS3_GFP, 3 µg of pCAGGS_T7, and 1 µg of p CAGGS_P3 were mixed with Opti-MEM^®^ I reduced-serum medium in the final volume of 150 µL. Fifty microliters of FuGENE^®^ HD was then added into the plasmids’ mixture, and the reaction was incubated at room temperature for 30 min to allow the complex formation. The transfection mixture was gently dripped onto the monolayer of BHK-21 cells prior to incubating the cells at 37 °C with 5% CO_2_ for 4 h. Thereafter, the transfection reactions were removed and replaced with either Opti-MEM^®^ I reduced-serum medium containing 50 µM (EC50) or 160 µM (EC90) (Theerawatanasirikul et al., a manuscript in preparation) of ribavirin (Sigma-Aldrich, Gillingham, UK). Then, the cells were incubated at 37 °C with 5% CO_2_. The fluorescent signals were observed at 24 hpt using a fluorescence microscope (Olympus, Center Valley, PA, USA). The signal intensity and numbers of green fluorescent cells were compared between drug and non-drug treated units. In addition, rupintrivir, a 3C^pro^ inhibitor, was tested at concentrations equal to its EC50 (2 µM) and EC90 (4 µM) (Theerawatanasirikul et al., a manuscript in preparation) to investigate the influence of protease inhibition on the RdRp function.

### 2.9. Molecular Docking of Ribavirin on FMDV RdRp

The interaction of ribavirin and FMDV O189 RdRp (3D^pol^) was studied by means of homology modeling exploiting the platform available on the SWISS-MODEL server (https://swissmodel.expasy.org/, accessed on 19 April 2021) [31] to elucidate the binding between ribavirin and O189 RdRp. The three-dimension structure of the reference 3D^pol^ (PDB code ID: 1wne.pdb, [32]) was used as the template for constructing a structural model of O189 3D^pol^. The quality of the modeled structure was analyzed by using Q-MEAN [33] and Ramachanadran plot [34]. The three-dimension structure of ribavirin (PubChem CID: 37542) was retrieved from the PubChem database for the molecular docking process. The protein structures were prepared as previously described [35]. The molecular docking was performed using PyRx 0.9.8 with Autodock Vina within the environment [36,37]. The docking grid center was randomly docked to the whole RdRp structure, and specified targets to the residues Pro44, Pro169, and M296 [38] with the grid box of x:20, y:20, z:20. The protein–ligand interaction was visualized using UCSF Chimera version 1.13.1 (UCSF, San Francisco, CA, USA) and BIOVIA Discovery Studio Visualizer 2017 v.12.0 (Dassault Systemes Biovia Corp, Waltham, MA, USA).

## 3. Results

### 3.1. Selection of the High Growth FMDV Strain

**Figure 2:**
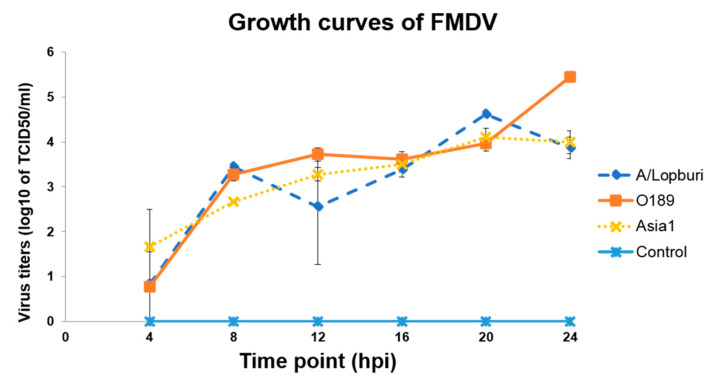
The growth kinetics of FMDV type A, A/Lopburi/2012, O/Tai/189/1987, and Asia1/2013 in BHK-21 cells at each time point. The cells were inoculated with each vaccine strain at 0.01 MOI. The viruses were collected for titration at 4-h intervals from 4 to 24 hpi. The bars represent the standard error of mean (SEM).

Growth characteristics of three FMDV strains, including type A; A/Lopburi/2012 (A/Lopburi), type O; O/TAI/189/1987 (O189), and type Asia1; Asia1/2013, in BHK-21 cells were determined. Cytopathic effects (CPE) characterized by cell rounding up and increasing reflection were first observed in the monolayers of cells infected with 0.01 MOI of each virus at approximately 12 hpi. The CPE produced by A/Lopburi/2012 was the most noticeable. The complete infected cell lysis appeared at 20 hpi. Viral growth curves were generated by plotting the virus titers against times (hpi). The results showed that the titers of all viruses continuously increased over the 24 h period of infection. FMDV O189 and A/Lopburi exhibited the highest growth characteristics (Figure 2). The overall analysis from the repeated measure ANOVA showed that log10 of mean titers of all viruses differed from that of non-virus control (*p* < 0.05). The inclusive titers of O189, Asia1, and A/Lopburi were similar. The analysis of the differences between mean titers at each time point is shown in Table S1. At the sixth time point (24 hpi), the mean titer of O189 was the highest and significantly higher than those of other isolates, with a *p* value less than 0.05. The titer of O189 was approximately 2 *×* 10^5^ TCID_50_/_mL_ at 24 hpi; therefore, it was selected for further study.

### 3.2. Components of pKLS3 Minigenome

**Figure 3:**
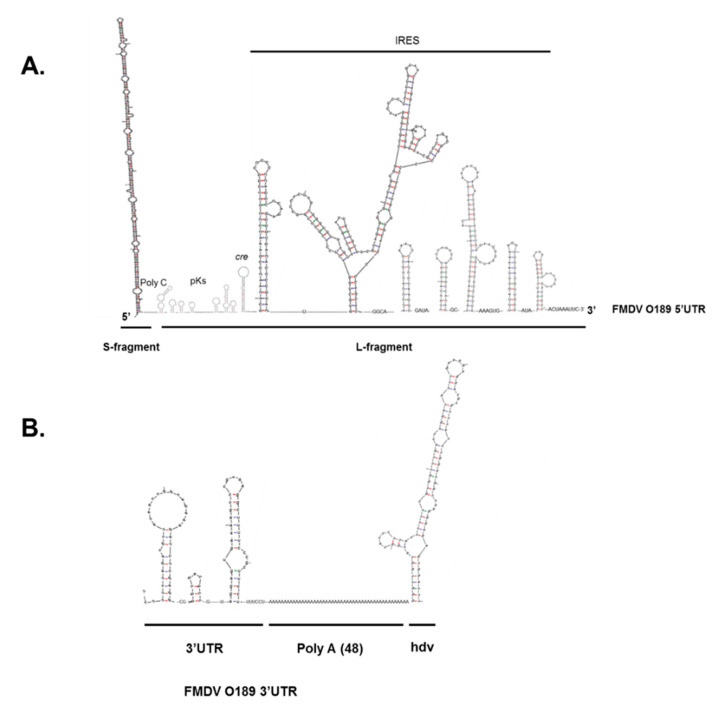
The regions of FMDV O189 UTRs and hdv ribozyme were used as the input sequences to generate the putative RNA secondary structures using mFold (RNA folding form version 2.3). (**A**) The 5′UTR comprised 368 nucleotides of S-fragment that formed a long stem–loop, followed by an l-fragment. The l-fragment was composed of 14 residues of a poly C, pseudoknots (pKs), cre, and IRES elements in a 5′ to 3′ direction. The pseudoknots spanned 175 nucleotides, while cre contained a conserve AAACA motif. The IRES spanned the rest of 5′UTR (located at 542–1053 nucleotides) upstream of the first start codon. (**B**) The 3′UTR contained 92 nucleotides folding into two stem–loop structures. Downstream the 3′UTR was a long poly A tail containing 48 adenine residues.

The FMDV minigenome, pKLS3, was constructed based on the essential cis-acting elements for viral replication and gene expression of FMDV O189. These elements included 5′UTR and 3′UTR with the poly A tail. The FMDV O189 5′UTR contained 1053 nucleotides which were arranged in 5′ to 3′ direction as an S-fragment (nucleotides 1-368), a stretch of 14 cytosine residues of the poly C tract, and an l-fragment (nucleotides 383-1053) (Figure 3A). The uridylylation initiation site for 3B, cre, of FMDV O189 located at nucleotides 558-661 contained a conserved motif AAACA at nucleotides 580–584. The putative secondary structures of FMDV O189 IRES comprised 7 stem–loop structures of 512 nucleotides (residues 542 to 1053), which theoretically form a complex tertiary structure for the ribosome binding. The 3′ UTR of FMDV O189 in the pKLS3 minigenome consisted of 92 nucleotides, which was followed by 48 adenine residues of the poly A tail (Figure 3B) and 85 nucleotides of the hepatitis delta virus (hdv) ribozyme, respectively. The cloning site for the FMDV coding sequence or foreign gene insertion was designed by introducing the recognition site for *Stu*I at the junction between O189 5′ and 3′UTRs. All these cis-acting elements—5′UTR, 3′UTR, and poly A tail—and the hdv ribozyme sequences were located downstream of the T7 RNA polymerase promoter (Figure 1A). Replication and transcription processes of the pKLS3 minigenome were driven by the T7 RNA polymerase promoter, while the translation process was initiated at the IRES once the uncapped mRNA was transcribed in the cytoplasm.

**Figure 4:**
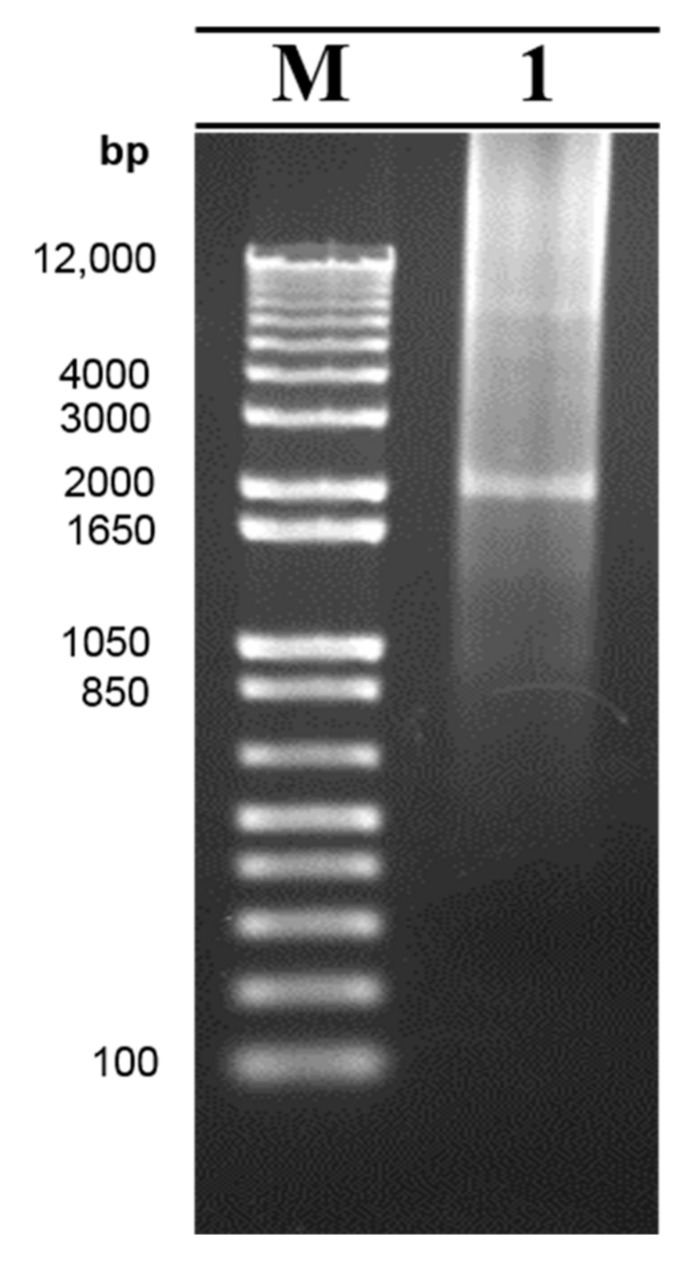
The electrophoretic photograph presenting an in vitro transcribed RNA containing the cis-acting elements of the pKLS3 minigenome and a GFP gene. The plasmid pKLS3_GFP was used as the plasmid DNA template in an in vitro transcription reaction to test the hdv ribozyme function. The hdv ribozyme auto-cleaved the transcribed RNA at its 5′ end, resulting in the RNA product containing the cis-acting elements of the pKLS3 minigenome and the GFP gene of approximately 2.2 kb (lane 1). Lane M is a standard molecular weight DNA marker.

The hdv ribozyme was included in the construct to free the transcribed RNA at its 5′ end. Thus, we tested the function of the ribozyme by an in vitro transcription using the closed-circular plasmid as the template. If it functioned properly, the hdv ribozyme was expected to auto-cleave precisely at its 5′ end. We found that the RNA product generated by the in vitro transcription was approximately 2.2 kb corresponding to the length of the whole O189 cis-acting elements and the hdv ribozyme presenting in the pKLS3 minigenome (Figure 4). The result suggests that the hdv ribozyme was able to cut the transcribed RNA.

### 3.3. Functional Evaluation of pKLS3 as An FMDV Minigenome

**Figure 5:**
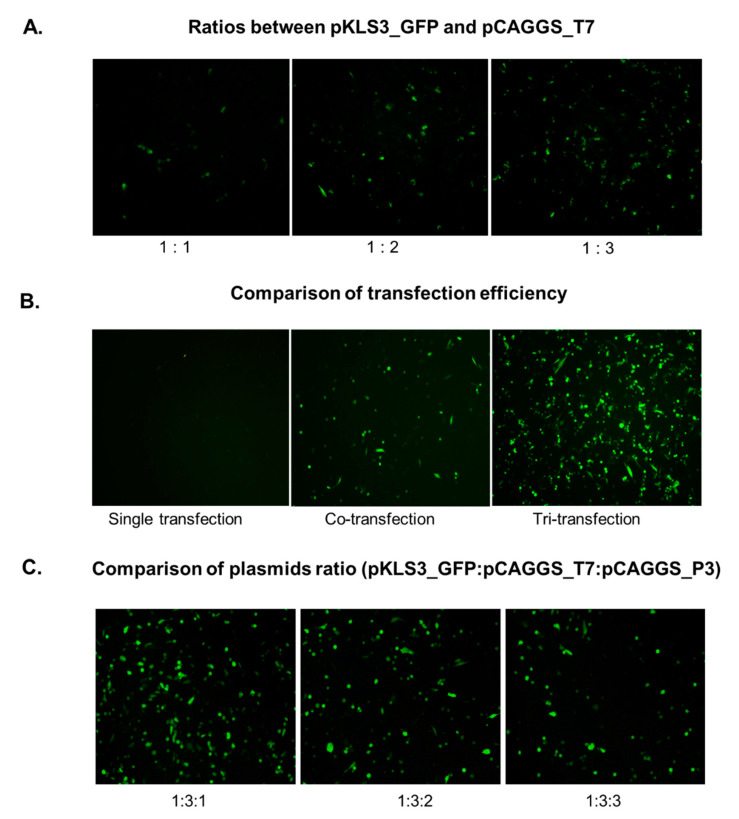
Photographs illustrating green fluorescent signals in transfected cells at x100 magnification. (**A**) The BHK-21 cells were co-transfected with pKLS3_GFP and pCAGGS_T7 at various ratios (pKLS3_GFP: pCAGGS_T7 = 1:1, 1:2, and 1:3). The *GFP* expression level was enhanced by the increased amount of the plasmid carrying T7 RNA polymerase, and the highest signal was observed when pCAGGS_T7 was three times pKLS3. (**B**) Tri-transfection with pKLS3_GFP, pCAGGS_T7, and pCAGGS_P3 dramatically elevated the *GFP* expression level compared to co-transfection with pKLS3_GFP and pCAGGS_T7. No detectable signal was observed in the single plasmid, pKLS3_GFP, transfected cells. (**C**) BHK-21 cells were tri-transfected with various ratios of pKLS3_GFP: pCAGGS_T7: pCAGGS_P3 (1:3:1, 1:3:2, and 1:3:3) and the optimum ratio was 1:3:1 as it produced the highest fluorescent signal.

To test the function of pKLS3 in transcription and translation processes, the *GFP* gene was introduced into pKLS3 at the *Stu*I site as a reporter gene to generate pKLS3_GFP (Figure 1B). In addition, pCAGGS_T7 was an indispensable helper plasmid, as it supplied intracellular T7 RNA polymerase in trans to transcribe the first strand RNA. The pKLS3_GFP and pCAGGS_T7 were directly co-transfected into BHK-21 cells at different plasmid ratios (pKLS3_GFP: pCAGGS_T7 = 1:1, 1:2, and 1:3). The green fluorescent cells were visualized in all transfection conditions at 24 hpt (Figure 5A). The *GFP* expression level was higher when increasing the amount of pCAGGS_T7. The highest fluorescent signals were detected at the pKLS3_GFP to pCAGGS_T7 ratio of 1 to 3, suggesting the optimal transfection condition (Figure 5A). Increasing the amount of pCAGGS_T7 from 3 to 4 µg did not improve the transfection efficiency in terms of fluorescent intensity and numbers of GFP positive cells. The results indicate that RNA molecules containing the *GFP* ORF were successfully translated into the GFP proteins clearly detected in transfected cells. However, the efficiency of *GFP* expression defined by the number GFP positive cells was less than 50%.

### 3.4. Enhancement of the GFP Expression Level by pCAGGS_P3

pCAGGS_P3 consisted of an ORF encoding for a P3 polyprotein of FMDV O189. Once translated, the P3 was processed to four mature proteins, including 3A, three copies of 3B (VPg), 3C (protease), and 3D (RdRp). This plasmid DNA was used as a helper vector to supply these proteins in trans. We expected that pCAGGS_P3 would expand the numbers of the transcripts produced by T7 RNA polymerase, serving as the templates for protein synthesis. The results demonstrated that the tri-transfection with pKLS3_GFP, pCAGGS_T7, and pCAGGS_P3 markedly increased both fluorescent signal intensity and numbers of the GFP expressing cells compared to the co-transfection with pKLS3_GFP and pCAGGS_T7 (Figure 5B). Additionally, the positive signal was not detected in culture wells transfected with pKLS3_GFP alone. We also found that increasing the pCAGGS_P3 ratio reduced the level of GFP expression (Figure 5C). Collectively, the results indicate that the optimal plasmid concentrations in the tri-transfection protocol were 1 µg of pKLS3_GFP, 3 µg of pCAGGS_T7, and 1 µg of pCAGGS_P3.

### 3.5. pKLS3_GFP as a Tool to Screen Antiviral Drug Targeting FMDV RdRp

The previous results showed that pCAGGS_T7 and pCAGGS_P3 could function in trans on the pKLS3 minigenome to drive transcription of the *GFP* gene. We then examined the application of this minigenome for antiviral drug screening. Ribavirin is a synthetic purine nucleoside (guanosine) analog with a broad-spectrum antiviral activity. It is effective against a large panel of RNA viruses, including picornaviruses, such as poliovirus, FMDV, and enterovirus 71. Inhibition of viral infection occurs through a direct interaction between RdRp and ribavirin-5′-triphosphate. The misincorporation of ribavirin into the viral genome results in increased viral mutation rates, leading to the extinction of the virus population [39]. In this study, the effect of ribavirin to inhibit the function of RdRp in the pKLS3 minigenome system was determined. In addition, we also investigated whether a non-RdRp inhibitor, rupintrivir, would influence our pKLS3_GFP minigenome system. Rupintrivir is a synthetic compound targeting the 3C^pro^ of picornaviruses, such as enterovirus 71 and human rhinovirus [40]. BHK-21 cells transfected with pKLS3_GFP, pCAGGS_T7, and pCAGGS_P3 were treated with either low (EC50) or high (EC90) doses of either ribavirin or rupintrivir. The cytotoxic effect of the tested antiviral compounds on BHK-21 cells was also determined, and the 50% cytotoxicity concentration (CC50) of ribavirin and rupintrivir were higher than 500 µM [35]. Thus, the drug concentrations used in this study had no impact on cell viability.

**Figure 6:**
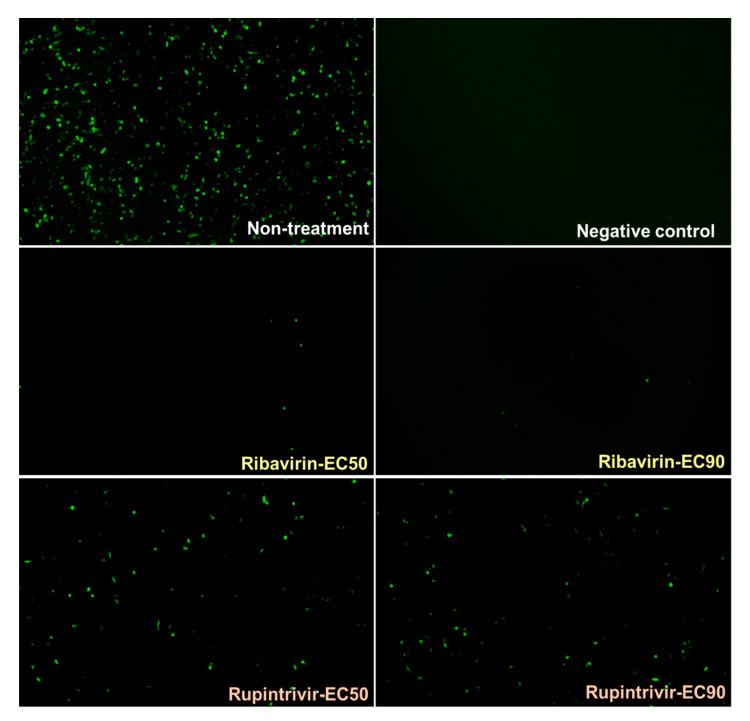
Application of the FMDV minigenome system composing of pKLS3_GFP, pCAGGS_T7, and pCAGGS_P3 in cell-based antiviral drug detection. BHK-21 cells were transfected with the three plasmids, then treated with antiviral agents, including ribavirin and rupintrivir, at EC50 and EC90 doses. Ribavirin inhibited FMDV RdRp leading to dramatically decreased GFP expression level, while rupintrivir significantly decreased the number of the GFP positive cells. The microscopic magnification was 100×.

The results showed that ribavirin almost completely inhibited the GFP expression as determined by markedly decreased numbers of GFP-positive cells (Figure 6). Rupintrivir, a 3C^pro^ inhibitor, which inhibits P3 processing, also decreased the GFP expression. However, the effect of the protease inhibitor was much less pronounced than that of ribavirin. The increased doses of ribavirin and rupintrivir from EC50 to EC90 did not decrease the fluorescent signals much. Please note that the EC50 and EC90 were performed using wild-type FMDV (Theerawatanasirikul et al., a manuscript in preparation). This finding indicates that our FMDV minigenome system using a combination of pKLS3_GFP and the two helper plasmids is a valuable screening tool for antiviral drugs targeting RdRp and may be 3C^pro^ of FMDV. Additionally, this platform can be applied to other picornaviruses.

### 3.6. Molecular Docking of Ribavirin on FMDV RdRp

**Figure 7:**
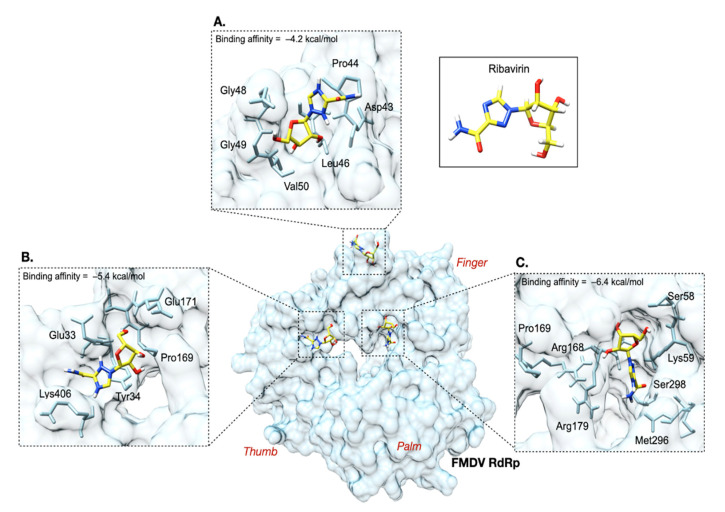
Molecular docking of ribavirin onto the three-dimension structure of FMDV O189 RdRp using AutoDock Vina algorithm in PyRx 0.9.8. The ribavirin molecule is displayed as a yellow stick. The backbone of ribavirin interactive residues within the RdRp is light blue. The insets show the binding of ribavirin to the potential residues. For specific docking, ribavirin reacted to Pro44 and Pro169 with the binding affinities of −4.2 kcal/mol (**A**) and −5.4 kcal/mol (**B**), respectively. In the random docking, ribavirin was preferentially buried in the deep pocket in which Met296 resided with the binding affinity of −6.4 kcal/mol (**C**). Notes: white, blue, and red sticks represent hydrogen, nitrogen, and oxygen, respectively.

The model of FMDV O189 RdRp was developed, and the quality of the structural modeling was adequate for the molecular docking analysis to confirm whether ribavirin could bind to the active site on RdRp of FMDV. The amino acid sequence identity between the reference and O189 RdRp was 98.94%. The structural quality determined by QMEAN was scored at −0.86, while the Ramachandran plot was favored at 94.23%. The results of randomized docking showed that the ribavirin molecule preferentially buried in the pocket containing the residues Pro169 and Met296 with a binding affinity of −5.4 and −6.4 kcal/mol, respectively (Figure 7). The residues Pro44, Pro169, and Met296, were selected as specific docking targets according to the previous study [38]. In their study, M296I, P44S, and P169S substitutions were observed in the viral populations after being consecutively passaged in the media with ribavirin. For specific docking, ribavirin mainly reacted to Pro44 via π-sigma (−4.2 kcal/mol) and Pro169 via van der Waals (−5.4 kcal/mol) interactions. The possible interlinkages and binding affinities of ribavirin on FMDV O189 RdRp are depicted in Figure 7.

## 4. Discussion

Viral replicon systems have been developed previously by a number of research groups to study the replication of RNA viruses. Replicons or minigenomes are defined as self-replicating but non-infectious RNAs. Thus, the FMDV minigenome provides an opportunity for molecular biology research related to FMDV replication, transcription, and translation, which has normally been restricted to high biosecurity and containment facilities. Here, we report the development of an FMDV minigenome, pKLS3, which is a DNA-based vector containing the minimum cis-acting elements essential for transcription and translation of FMDV. Because this platform utilizes T7 promoter to facilitate the transcription of the first-strand RNA, the pCAGGS_T7 plasmid expressing the T7 RNA polymerase is a crucial component in this system. Our minigenome possessed a hdv ribozyme downstream from the poly A tail, which auto-cleaved at its 5′end, leading to the transcription termination of the T7 RNA polymerase. Another component, pCAGGS_P3, is not mandatory; however, it acts as the transcription enhancer to increase the yield of the transcripts for protein synthesis by cap-independent translation. The ribosome assembled at the IRES located within the 5′UTR scans through the ribonucleotide sequence and starts reading at the first or second ATG to perform the translation process. Although various FMDV replicons or minigenomes were established, all required in vitro transcription and RNA transfection [23,24,25,26,41]. In addition, these minigenomes also contain the P2 sequence, which was absent in our construct. Thus, pKLS3 was the smallest FMDV minigenome thus far.

For pKLS3 to function as the minigenome, all cis-acting components for the transcription and translation processes, including 5′UTR and 3′UTR, should be intact and work properly. In fact, 5′UTR and 3′UTR interacted together and with other viral and host proteins to form a replication complex. Although the exact component of the replication complex has not been elucidated yet, evidence showed that the cellular poly C binding protein 2 (PCBP2) bound the IRES and interacted with the other host proteins, such as poly-A binding protein (PABP) that attached to the 3′UTR [26]. Translation efficiency also depends on the characteristics of 5′UTR, such as the length, the sequences upstream of the start codon, and the secondary structure, particularly IRES [42]. In addition, the 5′ and 3′UTRs contain binding sites for regulatory proteins [43] to facilitate both cap-dependent and cap-independent translation initiations through RNA interactions and helicase-mediated remodeling of RNA structures [44].

Although the helper plasmid containing the FMDV P3 polyprotein, pCAGGS_P3, was not a necessary component for the role of pKLS3 minigenome in the first strand RNA synthesis, it markedly enhanced the GFP expression. In addition to supply the RdRp for the efficient transcription of uncapped RNA produced by T7 RNA polymerase, the P3 also encodes a protease, 3C^pro^. FMDV 3C^pro^ is responsible for shutting off cellular transcription of host mRNA by cleaving the nuclear histone H3 and inhibiting host protein translation [45]. As cellular transcription occurs in the nucleus, 3C^pro^ provided in trans mostly interferes with the host protein synthesis by inhibiting cap-dependent translation initiation [46]. FMDV 3C^pro^ plays a significant role by cleaving eIF4AI and eIF4GI, the translation initiation factors required by capped mRNA for binding with the 40S ribosomal subunit and unwinding secondary structures of RNA [47]. Thus, the 3C^pro^ facilitated the translation of uncapped mRNA generated by our pKLS3 minigenome system by hijacking the cleaved eIF4GI for binding with its IRES.

The length of the poly A tail was highly important for the function of the pKLS3 minigenome. We found that the length of poly A tail was significantly related to the level of the GFP expression. One of our constructs containing 19 adenine residues failed to express a detectable level of the green fluorescent signal. Previous studies have shown that 3′UTR and the length of poly A tail of RNA viruses are responsible for viral genome replication [14,16]. In the poliovirus, the 10-fold increase in negative-strand RNA synthesis occurred by increasing the numbers of adenine from 12 to 13 residues [48]. In addition, increasing the poly A tail length from 15 to 20 residues has been shown to improve the viral copy number of duck hepatitis A virus type 1 [14]. In the hepatitis C virus, the poly A tail with 50 adenine residues demonstrated enhanced translation efficiency, depending upon its IRES [49]. A recent study related to mRNA vectors revealed that long poly A tails were essential for RNA stabilities, as they were naturally degraded in the cytoplasm [50]. In general, 30 adenine residues are needed for a functional mRNA, and the longer poly A tail, the higher mRNA stability [51]. These findings were consistent with our study, showing that the GFP expression in transfected cells was detected when the length of the poly A tail increased from 19 to 48 residues.

Recently, the mRNA vaccine has become one of the most robust technologies to produce vaccines and biomedical therapeutics for emerging infectious diseases and cancers due to its short manufacturing lead time and scalability. The transcribed mRNAs possess the same major advantages as DNA vaccines but lack the adverse effect of DNA integration into the host genome [52]. In addition, the process is more rapid and economical to produce than conventional vaccine production, as the viral or protein purification steps are not required. Since the final products do not contain any infectious agents as well as the by-products from mammalian cell culture, the mRNAs are considered safe. Mostly, mRNAs used for vaccines and drug therapies are generated by in vitro transcription using mRNA expression vectors carrying genes encoding the specific antigens [52,53]. In addition to the antigens, the cis-acting elements on the mRNA molecules should be properly designed for highly efficient protein synthesis in cells. Previously, the lacZ gene flanked by 5′ and 3′UTRs from *Xenopus laevis* β -globin was generated and used as an mRNA vaccine to immunize mice [52]. Furthermore, both 5′ and 3′UTRs could be optimized. For example, human endogenous genes were explored to design a platform for SARS-CoV-2 mRNA vaccine production [54]. In their study, the comparison between eukaryotes and non-eukaryotes UTRs revealed that the IRESs of encephalomyocarditis virus (EMCV) and FMDV possessed high binding affinities to ribosomal subunits and supported ribosomal retaining and recycling for more efficient translation. However, mRNA production is relatively challenging to maintain high quality and stability throughout the process.

Thus, the development of nucleic acid vaccines and therapeutics are mainly based on DNA molecules. Generally, a plasmid DNA, once delivered into the cells, still requires sequential numbers of intracellular events for DNA translocation from the cell periphery to the nucleus to initiate the transcription. The transcribed mRNA is then transported back to the cytoplasm and consecutively translated into a protein. Since plasmid DNA does not easily pass through the nuclear membranes, only a few DNA molecules can enter the nucleus even in actively dividing cells [55].

We have shown here for the first time the establishment of a DNA-based pKLS3 minigenome system and its application for foreign protein expression and antiviral drug screening. Although pKLS3 and the helper plasmids are DNA, both transcription and translation processes for efficient protein synthesis occur solely in the cytoplasmic compartment, such as the mRNA vaccine. Additionally, handling, storing, and transport of this minigenome system are more convenient than dealing with RNAs. Unlike some DNA and viral vector vaccines, they do not integrate into the host chromosome. With these properties, the pKLS3 minigenome could be one of the attractive candidate vectors for modern biopharma development.

## 5. Patents

pKLS3 vector, its associated products, and the developmental process are protected under the Patent Application Number: 1901006625.

## Figures and Tables

**Table 1:** Synthetic oligonucleotides used for amplification of FMDV 5′UTR.

Primers	Sequences (5′-3′)	Remark
FMDV_SF_F	TTGAAAGGGGGCGYTAGGGTYTCA	
FMDV_SF_R	GGGTGAGYRRGCYTCGG	
FMDV_polyC_F	TGGGCACTCCTGTTGGGG	
FMDV_polyC_R	TCCTCAAGCGACGGCG	
Mya98_LF_F	CCCCCCCCCCCCCYAAG	
Mya98_P1R	CWGCRGTGACTTCRACGTC	
KLS1_F1	TATTCTCGAGTTGAAAGGGGGCGCTAGGGT	Underlined: *Xho*I
KLS1_R1	GTGACATCTGAGGGAAGGCCTGAATTTAGTGGCAAT	Underlined: *Stu*I

**Table 2:** Sequences of primers used for cloning a *GFP* gene into pKLS3.

Primers	Sequences (5′-3′)	Remark
EGFP_F	ATTAAGGCCT**ATG**GTGAGCAAGGGCGAGGAGCTG	Underlined: *Stu*I Bold letter: start codon
EGFP_R	ATATAGGCCT**TTA**CTTGTACAGCTCGTCCATGCCGAG	Underlined: *Stu*I Bold letter: stop codon

**Table 3:** Sequences of primers used for cloning P3 ORF of FMDV O189.

Primer	Sequence (5′-3′)	Remark
ClaI_P3_F	ATCGATTT**ATG**ATCTCAATTCCTTCC	Underlined: *Cla*I Bold letter: start codon
NheI_P3_R	GCTAGC**TTA**YGCGTCACCRCA	Underlined: *Nhe*I Bold letter: stop codon

## Data Availability

No new data was created or analyzed in this study. Data sharing is not applicable to this article.

## Supplementary Materials

The followings are available online at https://www.mdpi.com/article/10.3390/v13061047/s1, Table S1: Comparison of the differences between mean ± standard deviation of log10 titers at each time point.
