# Supplementary material for: A Novel Plasmid DNA-Based Foot and Mouth Disease Virus Minigenome for Intracytoplasmic mRNA Production
Source: Viruses. 2021 Jun 1;13(6):1047. doi: 10.3390/v13061047 (PMC8229761; doi:10.3390/v13061047)
Supplement: Supplementary file 1 [file viruses-13-01047-s001.zip › viruses-1222504-Supplementary.pdf]

SUPPLEMENT DATA

SUPPLEMENTARY TABLE

Table S1: Comparison of the differences between mean ± standard deviation of log10 titers at each time point

Viruses	Mean ± SD of log10 titers at different time points (hours post inoculation)						Overall
	4	8	12	16	20	24	
O189	0.8^{a}±1.4	3.3^{a}±0.3	3.7^{a}±0.3	3.6^{a}±0.1	4.0^{b}±0.3	5.4^{a}±0.1	3.5^{a}±1.5
Asia I	1.7^{a}±1.4	2.7^{b}±0.0	3.3^{a}±0.3	3.5^{a}±0.5	4.1^{ab}±0.4	4.0^{b}±0.4	3.2^{a}±1.0
A/lopburi	0.8^{a}±1.4	3.4^{a}±0.1	2.6^{ab}±2.2	3.4^{a}±0.1	4.6^{a}±0.1	3.9^{b}±0.4	3.1^{ab}±1.5
O/41	2.5^{a}±0.0	2.5^{b}±0.0	2.5^{ab}±0.0	2.5^{b}±0.0	2.5^{c}±0.0	2.5^{c}±0.0	2.5^{b}±0.0
Non-virus control	0.0^{a}±0.0	0.0^{c}±0.0	0.0^{ab}±0.0	0.0^{c}±0.0	0.0^{d}±0.0	0.0^{d}±0.0	0.0^{c}±0.0

Note: The different letters over the mean titers at each time point indicate statistically significant differences.
